# Supplementary material for: TLRs Gene Polymorphisms Associated with Pneumonia before and during COVID-19 Pandemic
Source: Diagnostics (Basel). 2022 Dec 30;13(1):121. doi: 10.3390/diagnostics13010121 (PMC9818199; doi:10.3390/diagnostics13010121)
Supplement: Supplementary file 1 [file diagnostics-13-00121-s001.zip › Supplementary 1.pdf]

Supplementary S1. The comparison of allele frequencies against the EUR population for all studied SNPs

Samples	SNP	Minor allele, (MAF)	HWE*, *p*	Fisher Exact test for equality of allele frequencies, *p*
Case 1	rs5743551	G (0.243)	0.056	0.332
	rs5743708	A (0.033)	1.000	0.573
	rs3804100	C (0.072)	**0.040**	0.723
	rs4986790	G(0.079)	0.058	0.271
	rs5743810	A (0.368)	0.805	0.376
	rs1051730	T (0.316)	0.595	0.239
	rs3764880	G (0.274)	0.360**	0.903
Case 2	rs5743551	G (0.194)	0.074	**0.015**
	rs5743708	A (0.018)	1.000	0.786
	rs3804100	C (0.029)	1.000	0.110
	rs4986790	G (0.100)	0.588	**0.040**
	rs5743810	A (0.365)	0.643	0.310
	rs1051730	T (0.376)	0.487	0.864
	rs3764880	G (0.235)	0.519**	0.417
Control	rs5743551	G (0.232)	0.396	0.140
	rs5743708	A (0.030)	1.000	0.616
	rs3804100	C (0.081)	1.000	0.352
	rs4986790	G (0.091)	0.572	0.077
	rs5743810	A (0.333)	1.000	0.056
	rs1051730	T (0.338)	**0.013**	0.467
	rs3764880	G (0.213)	0.329**	0.150
EUR	rs5743551	G (0.285)	**0.016**	
	rs5743708	A (0.024)	1.000	
	rs3804100	C (0.064)	1.000	
	rs4986790	G (0.057)	0.207	-
	rs5743810	A (0.409)	0.461	
	rs1051730	T (0.369)	0.632	
	rs3764880	G (0.269)	0.566**	

*Haldane Exact test for Hardy-Weinberg equilibrium (autosomal) using SELOME p-value

**Graffelman-Weir exact test for Hardy-Weinberg equilibrium on the X-chromosome using SELOME p-value
