# Supplementary material for: TLRs Gene Polymorphisms Associated with Pneumonia before and during COVID-19 Pandemic
Source: Diagnostics (Basel). 2022 Dec 30;13(1):121. doi: 10.3390/diagnostics13010121 (PMC9818199; doi:10.3390/diagnostics13010121)
Supplement: Supplementary file 1 [file diagnostics-13-00121-s001.zip › Supplementary 2.pdf]

Supplementary S2. The comparison of genotype frequencies against the EUR population for all studied SNPs

SNP	Genotype	Samples	Genotype freq. in samples	Genotype freq. in EUR	χ^2 (*p*)
rs5743551	AA/AG/GG	Case 1	0.53/0.46/0.01	0.53/0.36/0.10	**7.52 (0.023)**
		Case 2	0.68/0.25/0.07		**6.58 (0.037)**
		Control	0.61/0.32/0.07		2.10 (0.350)
rs5743708	AG/GG	Case 1	0.07/0.93	0.05/0.95	0.15 (0.696)
		Case 2	0.035/0.965		0.05 (0.821)
		Control	0.06/0.94		0.08 (0.774)
rs3804100	CC/CT/TT	Case 1	0.03/0.09/0.88	0.004/0.1192/0.8767	5.19 (0.075)
		Case 2	0/0.06/0.94		3.08 (0.214)
		Control	0/0.16/0.84		1.71 (0.426)
rs4986790	AA/AG/GG	Case 1	0.87/0.10/0.03	0.8926/0.1014/0.006	3.22 (0.200)
		Case 2	0.81/0.18/0.01		4.55 (0.103)
		Control	0.83/0.16/0.01		3.30 (0.192)
rs5743810	AA/AG/GG	Case 1	0.14/0.45/0.41	0.17/0.47/0.36	0.86 (0.650)
		Case 2	0.11/0.49/0.39		1.73 (0.421)
		Control	0.11/0.44/0.44		3.81 (0.149)
rs1051730	CC/CT/TT	Case 1	0.45/0.47/0.08	0.40/0.46/0.14	2.28 (0.320)
		Case 2	0.36/0.52/0.12		1.18 (0.555)
		Control	0.49/0.33/0.17		5.00 (0.082)
rs3764880*	m: A/G<br>f: AA/AG/GG	Case 1	m: 0.70/0.30<br>f: 0.63/0.26/0.11	m: 0.71/0.29<br>f: 0.55/0.37/0.08	m: 0 (1)<br>f: 0.93 (0.630)
		Case 2	m: 0.81/0.19<br>f: 0.55/0.42/0.03		m: 0.48 (0.486)<br>f: 1.92 (0.382)
		Control	m: 0.875/0.125<br>f: 0.57/0.40/0.03		m: 2.14 (0.143)<br>f: 2,37 (0.306)

*m – male; f – female.
