# Supplementary material for: TLRs Gene Polymorphisms Associated with Pneumonia before and during COVID-19 Pandemic
Source: Diagnostics (Basel). 2022 Dec 30;13(1):121. doi: 10.3390/diagnostics13010121 (PMC9818199; doi:10.3390/diagnostics13010121)
Supplement: Supplementary file 1 [file diagnostics-13-00121-s001.zip › Supplementary 3.pdf]

<u>Supplementary S3. Comparison results of allele frequencies against Case 1, Case 2 and Control groups for all studied SNPs</u>

Table S1. Comparison results Case 1 and Control groups

SNP	Control	Allele 1 freq.	Case 1	Allele 2 freq.	OR CI95%	*p*
rs5743551						
Codominant						
A/A	60	60.6	40	52.6	1.00	0.04523
A/G	32	32.3	35	46.1	1.64(0.88-3.06)	
G/G	7	7.1	1	1.3	0.21(0.03-1.81)	
Dominant						
A/A	60	60.6	40	52.6	1.00	0.2909
A/G-G/G	39	39.4	36	47.4	1.38(0.76-2.53)	
Recessive						
A/A-A/G	92	92.9	75	98.7	1.00	0.05237
G/G	7	7.1	1	1.3	0.18(0.02-1.46)	
Overdominant						
A/A-G/G	67	67.7	41	53.9	1.00	0.06426
A/G	32	32.3	35	46.1	1.79(0.96-3.31)	
log-Additive						
0,1,2	99	56.6	76	43.4	1.07(0.64-1.78)	0.8032
rs5743708						
Codominant						
G/G	93	93.9	71	93.4	1.00	0.8888
A/G	6	6.1	5	6.6	1.09(0.32-3.72)	
log-Additive						
0,1,2	99	56.6	76	43.4	1.09(0.32-3.72)	
rs3804100						
Codominant						
T/T	83	83.8	67	88.2	1.00	0.1044
C/T	16	16.2	7	9.2	0.54(0.21-1.39)	
C/C	0	0.0	2	2.6	2.44 (0.29-131.0)	
Dominant						
T/T	83	83.8	67	88.2	1.00	0.4149
C/T-C/C	16	16.2	9	11.8	0.70(0.29-1.68)	
Recessive						
T/T-C/T	99	100.0	74	97.4	1.00	0.1872
C/C	0	0.0	2	2.6	2.64(032-141.2)	
Overdominant						
T/T-C/C	83	83.8	69	90.8	1.00	0.1706
C/T	16	16.2	7	9.2	0.53(0.20-1.35)	
log-Additive						
0,1,2	99	56.6	76	43.4	0.89(0.41-1.93)	0.1044
rs4986790						
Codominant						
A/A	82	82.8	66	86.8	1.00	0.4148
A/G	16	16.2	8	10.5	0.62(0.25-1.54)	
G/G	1	1.0	2	2.6	2.48(0.22-28.01)	
Dominant						

A/A	82	82.8	66	86.8	1.00	0.4636
A/G-G/G	17	17.2	10	13.2	0.73(0.31-1.70)	
Recessive						
A/A-A/G	98	99.0	74	97.4	1.00	0.4138
G/G	1	1.0	2	2.6	2.65(0.24-29.77)	
Overdominant						
A/A-G/G	83	83.8	68	89.5	1.00	0.2775
A/G	16	16.2	8	10.5	0.61(0.25-1.51)	
log-Additive						
0,1,2	99	56.6	76	43.4	0.87(0.42-1.79)	0.7077
rs5743810						
Codominant						
G/G	44	44.4	31	40.8	1.00	0.7715
A/G	44	44.4	34	44.7	1.10(0.58-2.08)	
A/A	11	11.1	11	14.5	1.42(0.55-3.68)	
Dominant						
G/G	44	44.4	31	40.8	1.00	0.628
A/G-A/A	55	55.6	45	59.2	1.16(0.63-2.13)	
Recessive						
G/G-A/G	88	88.9	65	85.5	1.00	0.5076
A/A	11	11.1	11	14.5	1.35(0.55-3.31)	
Overdominant						
G/G-A/A	55	55.6	42	55.3	1.00	0.9692
A/G	44	44.4	34	44.7	1.01(0.55-1.85)	
log-Additive						
0,1,2	99	56.6	76	43.4	1.16(0.75-1.81)	0.4989
rs3764880						
Codominant						
A/A	64	64.6	52	68.4	1.00	1.568×10^{-6}
A/G	30	30.3	5	6.6	0.21(0.07-0.57)	
G/G	5	5.1	19	25.0	4.68(1.64-13.38)	
Dominant						
A/A	64	64.6	52	68.4	1.00	0.6001
A/G-G/G	35	35.4	24	31.6	0.84(0.45-1.59)	
Recessive						
A/A-A/G	94	94.9	57	75.0	1.00	0.0001173
G/G	5	5.1	19	25.0	6.27(2.22-17.71)	
Overdominant						
A/A-G/G	69	69.7	71	93.4	1.00	4.133×10^{-5}
A/G	30	30.3	5	6.6	0.16(0.06-0.44)	
log-Additive						
0,1,2	99	56.6	76	43.4	1.36(0.90-2.06)	0.1438

Table S2. Comparison results Case 2 and Control groups

SNP	Control	Allele 1 freq.	Case 2	Allele 2 freq.	OR CI95%	*p*
rs5743551						
Codominant						
A/A	60	60.6	58	68.2	1.00	0.5106
A/G	32	32.3	21	24.7	0.68(0.35-1.31)	
G/G	7	7.1	6	7.1	0.89(0.28-2.80)	
Dominant						
A/A	60	60.6	58	68.2	1.00	0.2811
A/G-G/G	39	39.4	27	31.8	0.72(0.39-1.32)	
Recessive						
A/A-A/G	92	92.9	79	92.9	1.00	0.9975
G/G	7	7.1	6	7.1	1.00(0.32-3.09)	
Overdominant						
A/A-G/G	67	67.7	64	75.3	1.00	0.2538
A/G	32	32.3	21	24.7	0.69(0.36-1.31)	
log-Additive						
0,1,2	99	53.8	85	46.2	0.82(0.51-1.31)	0.4044
rs5743708						
Codominant						
G/G	93	93.9	82	96.5	1.00	0.4219
A/G	6	6.1	3	3.5	0.57(0.14-2.34)	
log-Additive						
0,1,2	99	53.8	85	46.2	0.57(0.14-2.34)	
rs3804100						
Codominant						
T/T	83	83.8	80	94.1	1.00	0.02465
C/T	16	16.2	5	5.9	0.32(0.11-0.93)	
log-Additive						
0,1,2	99	53.8	85	46.2	0.32(0.11-0.93)	0.02465
rs4986790						
Codominant						
A/A	82	82.8	69	81.2	1.00	0.9576
A/G	16	16.2	15	17.6	1.11(0.51-2.42)	
G/G	1	1.0	1	1.2	1.19(0.07-19.35)	
Dominant						
A/A	82	82.8	69	81.2	1.00	0.7711
A/G-G/G	17	17.2	16	18.8	1.12(0.53-2.38)	
Recessive						
A/A-A/G	98	99.0	84	98.8	1.00	0.9137
G/G	1	1.0	1	1.2	1.17(0.07-18.94)	
Overdominant						
A/A-G/G	83	83.8	70	82.4	1.00	0.7886
A/G	16	16.2	15	17.6	1.11(0.51-2.41)	
log-Additive						

0,1,2	99	53.8	85	46.2	1.11(0.56-2.21)	0.7695
rs5743810						
Codominant						
G/G	44	44.4	33	38.8	1.00	0.7391
A/G	44	44.4	42	49.4	1.27(0.69-2.36)	
A/A	11	11.1	10	11.8	1.21(0.46-3.19)	
Dominant						
G/G	44	44.4	33	38.8	1.00	0.4406
A/G-A/A	55	55.6	52	61.2	1.26(0.70-2.27)	
Recessive						
G/G-A/G	88	88.9	75	88.2	1.00	0.8895
A/A	11	11.1	10	11.8	1.07(0.43-2.65)	
Overdominant						
G/G-A/A	55	55.6	43	50.6	1.00	0.5007
A/G	44	44.4	42	49.4	1.22(0.68-2.18)	
log-Additive						
0,1,2	99	53.8	85	46.2	1.15(0.74-1.79)	0.5224
rs3764880						
Codominant						
A/A	64	64.6	52	61.2	1.00	0.808
A/G	30	30.3	27	31.8	1.11(0.59-2.09)	
G/G	5	5.1	6	7.1	1.48(0.43-5.11)	
Dominant						
A/A	64	64.6	52	61.2	1.00	0.627
A/G-G/G	35	35.4	33	38.8	1.16(0.64-2.11)	
Recessive						
A/A-A/G	94	94.9	79	92.9	1.00	0.5674
G/G	5	5.1	6	7.1	1.43(0.42-4.86)	
Overdominant						
A/A-G/G	69	69.7	58	68.2	1.00	0.8308
A/G	30	30.3	27	31.8	1.07(0.57-2.00)	
log-Additive						
0,1,2	99	53.8	85	46.2	1.16(0.72-1.88)	0.5398
